# Supplementary material for: A structural mechanism for phosphorylation-dependent inactivation of the AP2 complex
Source: eLife. 2019 Aug 29;8:e50003. doi: 10.7554/eLife.50003 (PMC6739873; doi:10.7554/eLife.50003)
Supplement: Supplementary file 1. [file elife-50003-supp1.docx]

**This supplementary file contains the following information associated with
‘*A structural mechanism for phosphorylation-dependent inactivation of the AP2 complex’***

*C. elegans* Strains

Recombinant proteins

DNA plasmids (including cloning strategies)

Sequences of synthesized gene fragments (IDT gBlocks)

Sequences of DNA oligos

***C. elegans Strains***

| **Wild-Type** | |
| --- | --- |
| Bristol N2 | *wild-type* |
| **Genetic screen parent strains** | |
| GUN61 | *mewSi1[Pdpy-30:apb-1(trunk):GFP:unc-54(3'UTR)] I; fcho-1(ox477::unc-119(+)) mewSi2[Pdpy-30:RFP:NCAP-1:unc-54(3'UTR)]*  *ncap-1(mew39[1.4 kb deletion]) II* |
| GUN141 | *fcho-1(mew55) ncap-1(mew54[ncap-1:mScarlet:3xFlag]) II* |
| **Genetic screen results** | |
| GUN294 | *fcho-1(mew55) ncap-1(mew54[ncap-1:mScarlet:3xFLAG]) II apm-2(mew156[T160A]) X* |
| GUN298 | *fcho-1(mew55) ncap-1(mew158[ncap-1(S84N):mScarlet:3xFLAG]) II* |
| GUN299 | *fcho-1(mew55) ncap-1(mew54[ncap-1:mScarlet:3xFLAG]) II apa-2(mew159[V469D]) X* |
| GUN300 | *fcho-1(mew55) ncap-1(mew54[ncap-1:mScarlet:3xFLAG]) II apb-1(mew160[S444P]) III* |
| GUN301 | *fcho-1(mew55) ncap-1(mew54[ncap-1:mScarlet:3xFLAG]) II apb-1(mew161[S444P]) III* |
| GUN302 | *fcho-1(mew55) ncap-1(mew54[ncap-1:mScarlet:3xFLAG]) II apb-1(mew162[S444P]) III* |
| GUN303 | *fcho-1(mew55) ncap-1(mew54[ncap-1:mScarlet:3xFLAG]) II apm-2(mew163[T160A]) X* |
| GUN304 | *fcho-1(mew55) ncap-1(mew54[ncap-1:mScarlet:3xFLAG]) II apm-2(mew164[T160A]) X* |
| GUN305 | *fcho-1(mew55) ncap-1(mew54[ncap-1:mScarlet:3xFLAG]) II apm-2(mew165[T160A]) X* |
| GUN306 | *fcho-1(mew55) ncap-1(mew54[ncap-1:mScarlet:3xFLAG]) II apm-2(mew166[T160A]) X* |
| GUN307 | *mewSi81[Pdpy-30:apb-1(trunk):GFP:unc-54(3'UTR)] I; fcho-1(ox477::unc-119(+)) mewSi2[Pdpy-30:RFP:NCAP-1:unc-54(3'UTR)] ncap-1(mew39[1.4 kb deletion]) apm-2(mew168[T160A]) X* |
| GUN308 | *mewSi81[Pdpy-30:apb-1(trunk):GFP:unc-54(3'UTR)] I; fcho-1(ox477::unc-119(+)) mewSi2[Pdpy-30:RFP:NCAP-1:unc-54(3'UTR)] ncap-1(mew39[1.4 kb deletion]) II apm-2(mew169[T160I]) X* |
| GUN309 | *mewSi81[Pdpy-30:apb-1(trunk):GFP:unc-54(3'UTR)] I; fcho-1(ox477::unc-119(+)) mewSi2[Pdpy-30:RFP:NCAP-1:unc-54(3'UTR)] ncap-1(mew39[1.4 kb deletion]) II apm-2(mew170[T160I]) X* |
| **Fitness assay and *in vivo* protease assay** | |
| *PHear Interface (Figures 2C and 2F)* | |
| GUN198 | *fcho-1(ox477::unc-119(+)) oxSi883[Phsp-16.41::TEVprotease unc-119(+)] ncap-1(mew39[1.4 kb deletion]) II; mewSi40[Pdpy-30:RFP:unc-54(3’UTR)] IV; apm-2(ox546[W64X]) oxSi877[Papm-2::3xFLAG:APM-2:tev-site unc-119(+)] X* |
| GUN106 | *fcho-1(ox477::unc-119(+)) oxSi883[Phsp-16.41::TEVprotease unc-119(+)] ncap-1(mew39[1.4 kb deletion]) II; mewSi3[Pdpy-30:RFP:NCAP-1:unc-54(3'UTR)] IV; apm-2(ox546[W64X]) oxSi877[Papm-2::3xFLAG:APM-2:tev-site unc-119(+)] X* |
| GUN296 | *fcho-1(ox477::unc-119(+)) oxSi883[Phsp-16.41::TEVprotease unc-119(+)] ncap-1(mew39[1.4 kb deletion]) II; mewSi3[Pdpy-30:RFP:NCAP-1:unc-54(3'UTR)] IV; apm-2(ox546[W64X]) mewSi79[Papm-2::3xFLAG:APM-2(T160A):tev-site unc-119(+)] X* |
| GUN135 | *fcho-1(ox477::unc-119(+)) oxSi883[Phsp-16.41::TEVprotease unc-119(+)] ncap-1(mew39[1.4 kb deletion]) II; mewSi35[Pdpy-30:RFP:NCAP-1(S84N):unc-54(3’UTR)] IV; apm-2(ox546[W64X]) oxSi877[Papm-2::3xFLAG:APM-2:tev-site unc-119(+)] X* |
| GUN252 | *fcho-1(ox477::unc-119(+)) oxSi883[Phsp-16.41::TEVprotease unc-119(+)] ncap-1(mew39[1.4 kb deletion]) II; mewSi65[Pdpy-30:RFP:NCAP-1(R109E):unc-54(3’UTR)] IV; apm-2(ox546[W64X]) oxSi877[Papm-2::3xFLAG:APM-2:tev-site unc-119(+)] X* |
| *Ex Interface (Figures 6C and 6E)* | |
| GUN198 | *fcho-1(ox477::unc-119(+)) oxSi883[Phsp-16.41::TEVprotease unc-119(+)] ncap-1(mew39[1.4 kb deletion]) II; mewSi40[Pdpy-30:RFP:unc-54(3’UTR)] IV; apm-2(ox546[W64X]) oxSi877[Papm-2::3xFLAG:APM-2:tev-site unc-119(+)] X* |
| GUN106 | *fcho-1(ox477::unc-119(+)) oxSi883[Phsp-16.41::TEVprotease unc-119(+)] ncap-1(mew39[1.4 kb deletion]) II; mewSi3[Pdpy-30:RFP:NCAP-1:unc-54(3'UTR)] IV; apm-2(ox546[W64X]) oxSi877[Papm-2::3xFLAG:APM-2:tev-site unc-119(+)] X* |
| GUN343 | *fcho-1(ox477::unc-119(+)) oxSi883[Phsp-16.41::TEVprotease unc-119(+)] ncap-1(mew39[1.4 kb deletion]) II; mewSi3[Pdpy-30:RFP:NCAP-1:unc-54(3'UTR)] IV; apa-2(mew191[V469D]) apm-2(ox546[W64X]) oxSi877[Papm-2::3xFLAG:APM-2:tev-site unc-119(+)] X* |
| GUN344 | *fcho-1(ox477::unc-119(+)) oxSi883[Phsp-16.41::TEVprotease unc-119(+)] ncap-1(mew39[1.4 kb deletion]) II; apb-1(mew192[S444P]) III; mewSi3[Pdpy-30:RFP:NCAP-1:unc-54(3'UTR)] IV; apm-2(ox546[W64X]) oxSi877[Papm-2::3xFLAG:APM-2:tev-site unc-119(+)] X* |
| GUN297 | *fcho-1(ox477::unc-119(+)) oxSi883[Phsp-16.41::TEVprotease unc-119(+)] ncap-1(mew39[1.4 kb deletion]) II; mewSi80[Pdpy-30:RFP:NCAP-1(K148A, E149A, G150A)] IV; apm-2(ox546[W64X]) oxSi877[Papm-2::3xFLAG:APM-2:tev-site unc-119(+)] X* |
| *Ex interface fitness assay (Figure 6C)* | |
| GUN87 | *ncap-1(mew54[ncap-1:mScarlet:3xFLAG]) II* |
| GUN88 | *ncap-1(mew54[ncap-1:mScarlet:3xFLAG]) fcho-1(ox477::unc-119(+)) II* |
| GUN291 | *ncap-1(mew54[ncap-1:mScarlet:3xFLAG]) fcho-1(ox477::unc-119(+)) II; apa-2(mew153[V469D]) X* |
| GUN288 | *ncap-1(mew54[ncap-1:mScarlet:3xFLAG]) fcho-1(ox477) II; apb-1(mew150[S444P]) III* |
| *NECAP structure-function (Figures 7A bottom and 7B)* | |
| GUN198 | *fcho-1(ox477::unc-119(+)) oxSi883[Phsp-16.41::TEVprotease unc-119(+)] ncap-1(mew39[1.4 kb deletion]) II; mewSi40[Pdpy-30:RFP:unc-54(3’UTR)] IV; apm-2(ox546[W64X]) oxSi877[Papm-2::3xFLAG:APM-2:tev-site unc-119(+)] X* |
| GUN106 | *fcho-1(ox477::unc-119(+)) oxSi883[Phsp-16.41::TEVprotease unc-119(+)] ncap-1(mew39[1.4 kb deletion]) II; mewSi3[Pdpy-30:RFP:NCAP-1:unc-54(3'UTR)] IV; apm-2(ox546[W64X]) oxSi877[Papm-2::3xFLAG:APM-2:tev-site unc-119(+)] X* |
| GUN276 | *fcho-1(ox477::unc-119(+)) oxSi883[Phsp-16.41::TEVprotease unc-119(+)] ncap-1(mew39[1.4 kb deletion]) II; mewSi75[Pdpy-30:RFP:NCAP-1(1-190)] IV; apm-2(ox546[W64X]) oxSi877[Papm-2::3xFLAG:APM-2:tev-site unc-119(+)] X* |
| GUN89 | *fcho-1(ox477::unc-119(+)) oxSi883[Phsp-16.41::TEVprotease unc-119(+)] ncap-1(mew39[1.4 kb deletion]) II; mewSi12[Pdpy-30:RFP:NCAP-1(1-163)] IV; apm-2(ox546[W64X]) oxSi877[Papm-2::3xFLAG:APM-2:tev-site unc-119(+)] X* |
| GUN195 | *fcho-1(ox477::unc-119(+)) oxSi883[Phsp-16.41::TEVprotease unc-119(+)] ncap-1(mew39[1.4 kb deletion]) II; mewSi37[Pdpy-30:RFP:NCAP-1(1-129)] IV; apm-2(ox546[W64X]) oxSi877[Papm-2::3xFLAG:APM-2:tev-site unc-119(+)] X* |
| GUN196 | *fcho-1(ox477::unc-119(+)) oxSi883[Phsp-16.41::TEVprotease unc-119(+)] ncap-1(mew39[1.4 kb deletion]) II; mewSi38[Pdpy-30:RFP:NCAP-1(130-236)] IV; apm-2(ox546[W64X]) oxSi877[Papm-2::3xFLAG:APM-2:tev-site unc-119(+)] X* |
| ***In vivo* imaging assay** | |
| *PHear interface (Figure 3E)* | |
| GUN204 | *mewSi81[Pdpy-30:apb-1(trunk):GFP:unc-54(3'UTR)] I; fcho-1(ox477::unc-119(+)) mewSi46[Pdpy-30:RFP:unc-54(3’UTR)] ncap-1(mew39[1.4 kb deletion]) II; apm-2(ox562[E306K])X* |
| GUN62 | *mewSi81[Pdpy-30:apb-1(trunk):GFP:unc-54(3'UTR)] I; fcho-1(ox477::unc-119(+)) mewSi2[Pdpy-30:RFP:NCAP-1:unc-54(3'UTR)] ncap-1(mew39[1.4 kb deletion]) II; apm-2(ox562[E306K])X* |
| GUN55 | *mewSi81[Pdpy-30:apb-1(trunk):GFP:unc-54(3'UTR)] I; fcho-1(ox477::unc-119(+)) mewSi2[Pdpy-30:RFP:NCAP-1:unc-54(3'UTR)] ncap-1(mew39[1.4 kb deletion]) II; apm-2(ox562[E302K]+mew46[T160A])X* |
| GUN123 | *mewSi81[Pdpy-30:apb-1(trunk):GFP:unc-54(3'UTR)] I; fcho-1(ox477::unc-119(+)) mewSi32[Pdpy-30:RFP:NCAP-1:unc-54(3'UTR)(S84N)] ncap-1(mew39[1.4 kb deletion]) II; apm-2(ox562[E306K])X* |
| GUN249 | *mewSi81[Pdpy-30:apb-1(trunk):GFP:unc-54(3'UTR)] I; fcho-1(ox477::unc-119(+)) mewSi62[Pdpy-30:RFP:NCAP-1:unc-54(3'UTR)(R109E)] ncap-1(mew39[1.4 kb deletion]) II; apm-2(ox562[E306K])X* |
| *Ex Interface (Figure 6D)* | |
| GUN204 | *mewSi81[Pdpy-30:apb-1(trunk):GFP:unc-54(3'UTR)] I; fcho-1(ox477::unc-119(+)) mewSi46[Pdpy-30:RFP:unc-54(3’UTR)] ncap-1(mew39[1.4 kb deletion]) II; apm-2(ox562[E306K])X* |
| GUN62 | *mewSi81[Pdpy-30:apb-1(trunk):GFP:unc-54(3'UTR)] I; fcho-1(ox477::unc-119(+)) mewSi2[Pdpy-30:RFP:NCAP-1:unc-54(3'UTR)] ncap-1(mew39[1.4 kb deletion]) II; apm-2(ox562[E306K])X* |
| GUN256 | *mewSi81[Pdpy-30:apb-1(trunk):GFP:unc-54(3'UTR)] I; fcho-1(ox477::unc-119(+)) mewSi2[Pdpy-30:RFP:NCAP-1:unc-54(3'UTR)] ncap-1(mew39[1.4 kb deletion]) II; apa-2(mew126[V469D]) apm-2(ox562[E306K])X* |
| GUN271 | *mewSi82[apb-1(trunk, S444P)] I; fcho-1(ox477::unc-119(+)) mewSi2[Pdpy-30:RFP:NCAP-1:unc-54(3'UTR)] ncap-1(mew39[1.4 kb deletion]) II; apb-1(mew138[S444P]) III; apm-2(ox562[E306K])X* |
| GUN253 | *mewSi1[apb-2(trunk):GFP] I; fcho-1(ox477::unc-119(+)) mewSi66[Pdpy-30:RFP:NCAP-1:unc-54(3'UTR)(K148A,E149A,G150A)] ncap-1(mew39[1.4 kb deletion]) II; apm-2(ox562[E306K])X* |

***Recombinant Proteins***

Recombinant protein sequences were derived from those below, with the addition of mutations, affinity tags, linkers, or other bioch­­emical tools.

Mouse AAK1 XP_006506255

Mouse AP2 beta1 NP_001030931

Mouse AP2 mu1 NP_033809

Mouse AP2 alpha2 NP_001343997

Rat AP2 sigma1 NP_075241.2

Human NECAP2 NP_060560

Mouse NECAP2 NP_079659

***DNA Plasmids***

| **NECAP expression plasmids** | |
| --- | --- |
| *Controls (Figure 1C, 3C, 4F, 5A, 6B, 7A top)* | |
| pGB86 | *HaloTag(TEVsite):6xHis in pET-21b* |
| pEP223 | *HaloTag(TEVsite):human NECAP2(1-263):6xHis in pET-21b* |
| *Structure Function (Figure 1C, 5A, 7A top)* | |
| pEP239 | *HaloTag(TEVsite):human NECAP2(1-195):6xHis in pET-21b* |
| pEP220 | *HaloTag(TEVsite):human NECAP2(1-170):6xHis in pET-21b* |
| pEP221 | *HaloTag(TEVsite):human NECAP2(1-132):6xHis in pET21b* |
| pEP241 | *HaloTag(TEVsite):human NECAP2(133-263):6xHis in pET21b* |
| *PHear Interface Mutants (Figure 3C)* | |
| pEP242 | *HaloTag(TEVsite):human NECAP2(S87N, 1-263):6xHis in pET-21b* |
| pEP245 | *HaloTag(TEVsite):human NECAP2(R112E, 1-263):6xHis in pET-21b* |
| *Ex Interface Mutants (Figure 6B)* | |
| pEP243 | *HaloTag(TEVsite):human NECAP2(K153A, E154A, G155A, 1-263):6xHis in pET-21b* |
| **AP2 expression plasmids** | |
| *Phospho-AP2 (Figure 1C, 3C)* | |
| pGH504 | *mouse AP2 alpha2(1-621):GST + rat AP2 sigma1(1-142) in pACYCduet-1* (Hollopeter *et al*., 2014) |
| pGB104 | *mouse AP2 beta1(1-591) + mouse AP2 mu1(1-435, thrombin site) in pETduet-1* |
| pEP82 | *mouse AAK1(1-325) in pRSFduet* (Beacham *et al*., 2018) |
| *Phospho-site mutant (Figure 3C)* | |
| pEP246 | *mouse AP2 beta1(1-591) + mouse AP2 mu1(T156A, 1-435, thrombin site) in pETduet-1* |
| *AP2 muE302K (Figure 4F, 5A, 6B)* | |
| pGH504 | *mouse AP2 alpha2(1-621):GST + rat AP2 sigma1(1-142) in pACYCduet-1* (Hollopeter *et al*., 2014) |
| pGB106 | *mouse AP2 beta1(1-591) + mouse AP2 mu1(E302K, 1-435, thrombin site) in pETduet-1* |
| *Ex interface mutants (Figure 6B)* | |
| pEP218 | *mouse AP2 alpha2(I470D, 1-621):GST + rat AP2 sigma1(1-142) in pACYCduet-1* |
| pEP213 | *mouse AP2 beta1(A447P, 1-591) + mouse AP2 mu1(E302K, 1-435, thrombin site) in pETduet-1* |
| *Phospho-AP2 muE302K (Figure 7A top)* | |
| pGH504 | *mouse AP2 alpha2(1-621):GST + rat AP2 sigma1(1-142) in pACYCduet-1* (Hollopeter *et al*., 2014) |
| pGB106 | *mouse AP2 beta1(1-591) + mouse AP2 mu1(E302K, 1-435, thrombin site) in pETduet-1* |
| pEP82 | *mouse AAK1(1-325) in pRSFduet* (Beacham *et al*., 2018) |
| **Plasmids for cryo-EM** | |
| *NECAP* | |
| pGH503 | *Mouse NECAP2(1-266):6xHis in pET-21b* (Beacham *et al*., 2018) |
| *Phospho-AP2 (unclamped, PDB 6OWO)* | |
| pGH504 | *mouse AP2 alpha2(1-621):GST + rat AP2 sigma1(1-142) in pACYCduet-1* (Hollopeter *et al*., 2014) |
| pGH419 | *mouse AP2 beta1(1-591) + mouse AP2 mu1(1-435) in pETduet-1* |
| pEP82 | *mouse AAK1(1-325) in pRSFduet* (Beacham *et al*., 2018) |
| *Closed AP2 (Figure 4D, 4E)* | |
| pGH504 | *mouse AP2 alpha2(1-621):GST + rat AP2 sigma1(1-142) in pACYCduet-1* (Hollopeter *et al*., 2014) |
| pGH419 | *mouse AP2 beta1(1-591) + mouse AP2 mu1(1-435) in pETduet-1* |
| *Open AP2 (Figure 4D, 4E)* | |
| pGH504 | *mouse AP2 alpha2(1-621):GST + rat AP2 sigma1(1-142) in pACYCduet-1* (Hollopeter *et al*., 2014) |
| pGB103 | *mouse AP2 beta1(1-591) + mouse AP2 mu1(E302K, 1-435) in pETduet-1* |
| *Phospho-AP2 (clamped, PDB 6OXL)* | |
| pGH504 | *mouse AP2 alpha2(1-621):GST + rat AP2 sigma1(1-142) in pACYCduet-1* (Hollopeter *et al*., 2014) |
| pGB103 | *mouse AP2 beta1(1-591) + mouse AP2 mu1(E302K, 1-435) in pETduet-1* |
| pEP82 | *mouse AAK1(1-325) in pRSFduet* (Beacham *et al*., 2018) |
| **Other plasmids** | |
| *MosSCI plasmid to generate PHearEx strain (Figure 7A bottom, Figure 7B)* | |
| pEP57 | *Pdpy-30:tagRFP-T:NCAP-1(1-163):unc-54(3’UTR) (cxTi10816 targeting)* |
| *PCR template to generate ncap-1:mScarlet CRISPR repair template* | |
| pSEM87 | *twk-18:mScarlet CRISPR repair* (El Mouridi *et al*., 2017) |

**Cloning of Plasmids**

The plasmids below were generated through mutation of an existing plasmid by PCR amplification of the template followed by Gibson assembly (one piece). See oligo list for sequences of PCR oligos.

Plasmid Template PCR Oligos

pEP213 pGB106 oEP973/oEP974

pEP218 pGH504 oEP977/oEP978

pEP220 pEP223 oEP1031/oEP1034

pEP221 pEP223 oEP1031/oEP1035

pEP239 pEP223 oEP1051/oEP1031

pEP241 pEP223 oEP1032/oEP1036

pEP242 pEP223 oEP1010/oEP1011

pEP243 pEP223 oEP1041/oEP1042

pEP244 pGB106 oGB24/oGB33

pEP245 pEP223 oEP1037/oEP1038

pEP246 pGB104 oGB24/oGB33

pGB86 pGB76 oGB175/oGB180

pGB103 pGB51 oGB172/oGB173

pGB104 pGH419 oGB34/oGB35

pGB106 pGB103 oGB34/oGB35

pGH419 pGH424 oGH367/oGH369

The plasmids below were generated through Gibson assembly reactions comprising multiple fragments. The strategy is detailed for each plasmid.

pEP1

The backbone of pET21b was amplified by PCR using oligos oEP318/oEP324. The resulting PCR product was combined with IDT gBlock gbEP11 in a Gibson assembly reaction, yielding pEP1.

pEP57

The MosSCI targeting plasmid backbone for *cxTi10816*, including the *C. elegans dpy-30* promoter, an N-terminal tagRFP-T and the *C. elegans unc-54* 3’UTR was amplified by PCR from pGH486 using oligos oGH1011/oGH1012. The DNA encoding amino acids 1-163 of *C. elegans* NECAP was amplified by PCR from cloned cDNA using oligos oEP366/oEP369. These two PCR products were combined in a Gibson assembly reaction, yielding pEP57.

pEP223

The pET-21b backbone along with a TEV cleavable HaloTag was amplified by PCR from pGB63 using oligos oEP1031/oEP1032. The DNA encoding amino acids 1-170 of human NECAP2 was amplified by PCR from pEP1 using oligos oEP1033/oEP641. These two PCR products were combined with IDT gBlock gbEP42 in a Gibson assembly reaction, yielding pEP223.

**Sequences of synthesized gene fragments**

gbEP11: Human NECAP2(1-170) for pET-21b

TTTAAGAAGGAGATATACATATGGAAGAAAGCGGCTATGAAAGCGTTCTGTGTGTTAAACCGGATGTTCATGTTTATCGTATTCCGCCTCGTGCAACCAATCGTGGTTATCGTGCAGCAGAATGGCAGCTGGATCAGCCGAGCTGGTCAGGTCGTCTGCGTATTACCGCAAAAGGTCAGATGGCATATATCAAACTGGAAGATCGTACCAGCGGTGAACTGTTTGCACAGGCACCGGTTGATCAGTTTCCGGGTACAGCAGTTGAAAGCGTGACCGATAGCAGCCGTTATTTTGTTATTCGTATTGAAGATGGTAATGGTCGCCGTGCCTTTATTGGTATTGGTTTTGGTGATCGTGGTGATGCCTTTGATTTTAATGTTGCACTGCAGGATCACTTTAAATGGGTTAAACAGCAGTGCGAATTTGCAAAACAGGCACAGAATCCGGATCAGGGTCCGAAACTGGATCTGGGTTTTAAAGAAGGTCAGACCATCAAACTGAACATTGCCAACATGAAAAAAAAAGAAGGCCATCATCACCATCACCACTAACCGCTGAGCAATAACTAGCATAACCC

gbEP42: Human NECAP2(171-263) for pET-21b

AACATGAAAAAAAAAGAAGGCGCAGCGGGTAACCCCCGTGTACGTCCGGCGTCTACTGGCGGCCTTTCCCTTTTGCCTCCACCACCGGGTGGGAAAACCTCTACACTTATTCCTCCACCAGGGGAACAACTGGCGGTAGGTGGCTCTTTGGTTCAGCCTGCTGTGGCCCCTAGCAGTGGTGGTGCTCCAGTACCTTGGCCCCAACCGAACCCCGCTACCGCGGATATCTGGGGAGACTTCACCAAGTCCACGGGCTCTACCTCAAGTCAGACGCAGCCAGGCACGGGCTGGGTACAATTTCATCACCATCATCACCACTGA

**Sequences of DNA oligos**

| **Oligo ID** | **SEQUENCE** |
| --- | --- |
| oEP318: | CATCATCACCATCACCACTAA |
| oEP324: | ATGTATATCTCCTTCTTAAAGTTAAACAAAATTAT |
| oEP366: | AACGGGCGGTAGTGGAGGCACTGGTATGGGAGATTACGAGAACGTTTTAATG |
| oEP369: | TATCACCACTTTGTACAAGAAAGCTGGGTCTAACTTTTATCCTTTTTTCCAATGTTAATT |
| oEP512: | AGTACTAGCGGTGGCAGTGGAGGTACCGGCGGAAGCATGGTCAGCAAGGGAGAGGCAGTT |
| oEP513: | CCGTCTTTATAGTCACCATCGTGGTCTTTGTAGTCCTTGTAGAGCTCGTCCATTCCTCCG |
| oEP519: | TTCCGACTAAAAATCCCCAAATTTTCAGAGATTTCAGTACTAGCGGTGGCAGTGGAGGTA |
| oEP520: | AGTTGTACGGAGAAGAAAGATGACGTCATCGCTTATCCTCCTTTGTCGTCATCATCCTTA |
| oEP582: | GGAGGAACGGGCGGTAGTGGAGGCACTGGTTAGACCTAGCTTTCTTGTACAAAGTGGTGA |
| oEP584: | GGAGGAACGGGCGGTAGTGGAGGCACTGGTGCGGAACTGGAAAAACAGGATCTTTCTGCC |
| oEP641: | GCCTTCTTTTTTTTTCATGTTGG |
| oEP655: | AAAAAGTCGATAGAGAAGGCTTCAACACAC |
| oEP656: | TCGGTCAAATTTCCCGGTTTTTAAC |
| oEP657: | ACCCGATTTTCTCGGTTTTTCTCTC |
| oEP659: | ATAAAAGTACGGATTTTTGCTCGAAATCAAC |
| oEP660: | GGCCAATTTTGAGGATTCTTTGGC |
| oEP661: | TTTAGACTGAAAATTCCGATTTTTGAGCC |
| oEP662: | GGTGGAGAGAGAGAAGTGAAGAGACGC |
| oEP670: | GCAAACTGGGGCACAAACTTAATTCC |
| oEP793: | GATCACTTTCGTTATATCGAACGAAGCTAATTCTTGTACAAAGTGGTGATATCTGAGCTC |
| oEP808: | AACAACATGAAGTGGCAGTCGC |
| oEP812: | GGAGCACAGGGAGAAAGAGC |
| oEP894: | AGGCATTCGTGGGATGCGGGTTTCAAGAAGAGGGAGATGCTTTTGACTTTAATGTCACAC |
| oEP865: | GGATGCGGGTTTCAAGAAGAG |
| oEP969: | CGTCAAGTGAAGCTGATCCGTGGCGTAGTTGGCTCACTTGGATTAATCGTAATCCC |
| oEP970: | GGGATTACGATTAATCCAAGTGAGCCAACTACGCCACGGATCAGCTTCACTTGACG |
| oEP971: | CGTCAAGTGAAGCTGATCCGTGGCGTAGTTGGCTCACTTGGATTAATCGTAATCCCGCTA |
| oEP972 | TAGCGGGATTACGATTAATCCAAGTGAGCCAACTACGCCACGGATCAGCTTCACTTGACG |
| oEP973: | TGCTCGAGCACCTATGATTTGGATTGTAGGAGAATATGC |
| oEP974: | TAGGTGCTCGAGCATCGGGTTCATCCAG |
| oEP977: | ATTGTCGACAATCGCGATGATGTGCAG |
| oEP978: | CGATTGTCGACAATCTGGATGACGCG |
| oEP985: | TGCCGGTCCAAGCTTGGATTTAGCATTTGCAGCTGCACAAACCATCTCAATTAACATTGG |
| oEP987: | GTTATTATTTCGGATTGTCGACAATAGGGAGGACGTTCAAGGATACGCAGCAAAGACTGT |
| oEP1010: | ACCGATAACAGCCGTTATTTTGTTATTCGTA |
| oEP1011: | ACGGCTGTTATCGGTCACGCT |
| oEP1014: | GGGACAAACCATCTCAATTAACATTGGAAAAAAAGACAAGTCATAGACTCAGCTTTCTTGTACAAAGTGGTGATATCTGA |
| oEP1016: | CAGTGAAAAGTTCTTCTCCTTTACT |
| oEP1020: | TAGTGCCACTGCTTCCACCGCCGCCAGGTGCCGGCTAAACCCAGCTTTCTTGTACAAAGT |
| oEP1031: | CATCACCATCATCACCACTGAGATC |
| oEP1032: | CATGCTTCCGCCGGTAC |
| oEP1033: | AGGTACCGGCGGAAGCATGGAAGAAAGCGGCTATG |
| oEP1034: | TCAGTGGTGATGATGGTGATGGCCTTCTTTTTTTTTCATGTTGGC |
| oEP1035: | TCAGTGGTGATGATGGTGATGCTGCTGTTTAACCCATTTAAAGTG |
| oEP1036 | GGTACCGGCGGAAGCTGCGAATTTGCAAAACAGGC |
| oEP1037: | TGATGAGGGTGATGCCTTTGATTTTAATG |
| oEP1038: | CACCCTCATCACCAAAACCAATACCAAT |
| oEP1041: | TTTTGCAGCAGCTCAGACCATCAAACTGAACATTG |
| oEP1042: | CTGAGCTGCTGCAAAACCCAGATCCAGTTTCGG |
| oEP1051: | TCAGTGGTGATGATGGTGATGTTTCCCACCCGGTGGTG |
| oGB24: | CGCCGCCAGCCAATCTGCCCAGCCACCTGGCTGGTGATCTGGGACTGTTC |
| oGB26: | ATGAATAAGCCTCCGATCATCATATGTATATCTCCTTCTTATA |
| oGB27: | GCATTTATGAAACCCGCTGCTAATTAACCTAGGCTGCTGCCACCG |
| oGB28: | ATGATCGGAGGCTTATTCATCT |
| oGB29: | GCAGCGGGTTTCATAAATGCCA |
| oGB33: | GGGCAGATTGGCTGGCGGCGAGAAGGCATCAAGTA |
| oGB34: | AGCAAGAGTCTGGTGCCGCGCGGCAGCGGA |
| oGB35: | CTGCTTACCGCTGCCGCGCGGCACCAGACCTTGCTTGTTTCATCAGCTGTG |
| oGB52: | TGCATCACGGGAGATGCACT |
| oGB124: | GGCTGGGTCCAGTTCCATCACCATCATCACCACTGA |
| oGB125: | GAACTGGACCCAGCCGGTGC |
| oGB130: | GGAGCAGTCACAAATCACGTCTCAAGTTGCCGGCCAAATTGGATGGCGTCGGGAGGGTAT |
| oGB147: | ATCTCCCGTGATGCAGGGCCTGGCTCTTGGGGTAC |
| oGB148: | TGTGAGTTTGCGAAACAAGC |
| oGB149: | TTTCGCAAACTCACACATGCTTCCGCCGGTACCTC |
| oGB172: | ATGATCGGAGGCTTATTCATCT |
| oGB173: | TAGATGAATAAGCCTCCGATCATATGTATATCTCCTTCT |
| oGB175: | CATCACCATCATCACCAC |
| oGB180: | CAGTGGTGATGATGGTGATGGCTTCCGCCGGTACCTCCAC |
| oGH367: | TAATGCTTAAGTCGAACAGAAAGTAATCG |
| oGH369: | TCTGTTCGACTTAAGCATTATTTGCGATGAATCCCATGAC |
| oGH408: | GCATTTTTCACATTTTCTAACATTTTTTCTGTTGAAAAG |
| oGH409: | GCACATTTTAAGTCTGTAAAAGTGAAAACCCA |
| oGH411: | TCCTATGCTCAGTCAGTGTATGAGC |
| oGH412: | GCTTTTGGAGCATTTTGTTTTCTAATTTTGAATGA |
| oGH413: | AAGTTTTATACCAAGTTTAGAACATGGATTCGG |
| oGH414: | CGGTTCTGCATGCAGTTGTCTG |
| oGH415: | CCAAAAAAATGTATCTGAATAAGTAAAGCAAAGTGATTC |
| oGH416: | GTCTTAACCAAAGAGCAACAACAATACCT |
| oGH417: | ACTATAACTTTTGATTGTTTTGTCAACAGCTAGC |
| oGH418: | CCACTTTTTCTAATATTTCAAACTTGTGCTCGA |
| oGH419: | TGTAAAAGTGGAGAGATGGCACGG |
| oGH430: | GTTTTGCAAAGATATTTTAATGAAGTTTGGCTCA |
| oGH432: | AAATTAATTGTTTCTACAGAGTGTTTCAATGTTTGAAC |
| oGH441: | GCTCCAATTTCCTTGAAACCTCG |
| oGH442: | CCTTGAAAGCTTTTTTTAAGTTTTTTAGGTG |
| oGH443: | GATTTTTCAAAATTTTTAACATCGAAACTCCC |
| oGH444: | GCCCGATTTTACAGGAACTCC |
| oGH445: | CTAAAATTCTAAACTACAAAATAATAATAAAAATATC |
| oGH446: | TGCAATTTTTACAGGTCAGG |
| oGH447: | CTCGGAAATTCAAATTATACATCAAAAATTATCAC |
| oGH448: | GAAATTCAGAATTATTTAGGGGAAAAGGC |
| oGH452: | CCATTCATATTTTGTCTCAGGAGAATAC |
| oGH679: | AGGTATTCAGACATTTTTCAAATGAAAATCTAC |
| oGH847: | CCAAACTGAAGGTCAAGGTGGTC |
| oGH848: | CCTTGACCTTCAGTTTGGTGCGC |
| oGH1014: | CCGCCGTCGTTCTCTCCACCG |
| RWB099: | GGCCTCCTTCGTCGTCTTCAGGATCCAATTCGAGCTCGAACAACAAC |
